# Seasonal Variation in the Thermoregulation Pattern of an Insular Agamid Lizard

**DOI:** 10.3390/ani13203195

**Published:** 2023-10-13

**Authors:** Emmanouela Karameta, Ioanna Gavriilidi, Spyros Sfenthourakis, Panayiotis Pafilis

**Affiliations:** 1 Section of Zoology and Marine Biology, Department of Biology, National and Kapodistrian University of Athens, Panepistimiopolis, Ilissia, 15784 Athens, Greece; ioanna.gavriilidi@uantwerpen.be (I.G.); ppafil@biol.uoa.gr (P.P.); 2 Department of Biology, University of Antwerp, Universiteitsplein 1, B-2610 Wilrijk, Belgium; 3 Department of Biological Sciences, University of Cyprus, Panepistimiou 1, 2109 Nicosia, Cyprus; sfendour@ucy.ac.cy; 4 Zoological Museum, National and Kapodistrian University of Athens, Panepistimioupolis, 15784 Athens, Greece

**Keywords:** seasonality, thermoregulation, islands, Agamidae

## Abstract

**Simple Summary:**

The ability of animals to maintain their body temperature within an optimal range, known as thermoregulation, is essential for their survival, overall health, and daily activities. Ectotherms, including reptiles, rely on external energy resources to regulate their body temperature. How well they can achieve this, heavily depends on various environmental factors, such as the climate and its seasonal changes. Islands typically have a mild climate, which is expected to favor the thermoregulation of reptiles throughout the year. In this study, we investigate the effect of seasonality on the thermoregulation efficiency and behavior of a population of lizards found on Naxos Island, in the Cyclades, Greece. Our results reveal that seasonal fluctuations significantly influence how easily and precisely lizards can regulate their body temperature, with summer being the most favorable period, and autumn being the least favorable. Interestingly, lizards adjusted their thermal preferences and thermoregulation efficiency depending on the challenges imposed by each season and thus managed to maintain stable body temperatures. Whether these adjustments represent evolutionary adaptations or simply reversible shifts, awaits further research. Understanding how lizards adapt to their changing environment can provide valuable insights into their survival strategies and how they may cope with future environmental changes.

**Abstract:**

Ectotherms, including lizards, rely on behavioral thermoregulation to maintain their body temperature within an optimal range. The benign climate of islands is expected to favor the thermoregulation efficiency of reptiles throughout their activity period. In this study, we investigated the seasonal variation in thermoregulation in an insular population of the roughtail rock agama (*Laudakia stellio*) on Naxos Island, Greece. We measured body, operative, and preferred temperatures across three seasons (spring, summer, and autumn), and we evaluated the effectiveness of thermoregulation, using the Hertz index (E). Our results revealed that the effectiveness of thermoregulation was significantly influenced by seasonality. E was quite high in summer (0.97) and spring (0.92), and lowest in autumn (0.81). Accordingly, the quality of the thermal environment was significantly low during autumn, and maximum during summer. However, despite the environmental temperature fluctuations, lizards exhibited remarkable stability in body temperatures. They also adjusted their preferred temperatures seasonally and doubled the thermal niche breadth they occupied during summer, thus enhancing thermoregulation efficiency. Whether or not these adjustments are plastic or fixed local adaptations remains to be explored in further research across multiple years and seasons, including additional insular populations.

## 1. Introduction

Ectothermic vertebrates regulate their body temperature (Tb) using energy stemming from external, environmental sources, in contrast to endotherms that rely mainly on metabolic heat [1,2]. The former, predominantly fish, amphibians, and reptiles, employ behavioral mechanisms to control their Tb, while the latter, mainly birds and mammals, use both behavioral tactics as well as changes in autonomic effector activity, such as shivering, skeletal muscle thermogenesis, etc. [3]. Regardless of the category they belong to, animals try to maintain their Tb within an optimal range, a process known as active thermoregulation [4]. In reptiles and amphibians, thermoregulation includes behavioral responses (basking, retreating to shaded microhabitats, underwater submersion, etc.), allowing animals to gain or lose heat via convection, conduction, radiation, and evaporation [5,6]. On the other hand, very few large-bodied reptile species with low metabolism, such as the Leatherback Sea Turtles (*Dermochelys coriacea*), can maintain constantly high Tb in comparison to the surrounding environmental temperatures, due to inertial endothermy (gigantothermy) [7]. In contrast, the much smaller tegu lizard (*Salvator merianae*) can achieve facultative endothermy via metabolic thermogenesis and decreased thermal conductance during the reproductive season [8].

The ability of ectotherms to keep their Tb within or close to an optimum range is imperative for their survival, condition, as well as daily activities, and performance, such as locomotion, foraging ability, growth rate, and reproductive investment [5,9]. At the individual level, the efficiency of thermoregulation is constrained by the trade-off between the costs and benefits it entails: intraspecific competition and predation risk on one hand, and the maximizing of performance and fitness on the other [4,10,11]. At the environmental level, it heavily depends on the availability of operative temperatures and spatial heterogeneity of a particular habitat [12].

Seasonal variations in environmental conditions, such as rainfall, wind intensity, temperature, and sunlight exposure may influence many of the behavioral and physiological attributes of lizards [13,14], including thermoregulation [15,16]. For example, subtropical lizards may shift their activity and thermoregulation effort as a response to weather fluctuations but are sensitive to extreme winter conditions [17]. Likewise, temperate species can change their thermoregulatory behavior, including daily activity and microhabitat selection, depending on the season [18], or even shift their preferred temperature range [19] in an effort to maximize thermoregulation effectiveness. Besides, a compilation of meta-analyses examining the effect of various factors on thermoregulation, such as climate, body size, habitat, altitude, season, and insularity, concluded that the most important ones were altitude and seasonality [20]. 

In this study, we aim to examine the seasonal variation in the effectiveness of thermoregulation in an insular population of the roughtail rock agama (*Laudakia stellio*), the only agamid ranging in Europe (Aegean islands, Greece). The challenges posed by seasonal variation may be buffered by island climate conditions that differ from those of the mainland [21,22]. This more benign insular climate is reflected in higher-quality thermal habitats that lower the thermoregulatory effort made by lizards [23,24]. However, small islets deviate from this general pattern. Due to their limited heterogeneity [25], they are thermally demanding habitats that may promote high thermoregulation effectiveness [26,27,28]. To avoid islet particularities, we focused on a population inhabiting the largest Cycladic Island, Naxos (430 km^2^). Just a handful of studies have assessed the effect of thermoregulatory seasonality on Mediterranean lizards [15,18,29,30] and none has focused on a large island. Here we posed a simple question: do the favorable insular environmental conditions minimize seasonal variations in thermoregulation? If the insular climate is indeed milder, there should be no seasonal variation in thermoregulation; if not, lizards should shift their thermoregulatory effectiveness in response to weather changes.

## 2. Materials and Methods

### 2.1. Study System

*Laudakia stellio* (Linnaeus, 1758) is a diurnal agamid lizard measuring up to 133 mm (maximum snout-vent length, SVL) [31]. It is widespread in the E. Mediterranean, where it is found mostly in rocky habitats and human-made constructions, in arid and semi-arid parts of Turkey and Greece [32]. It can live up to 7 years in the wild [33] and females lay 4–10 eggs from May until July [34]. It employs a combination of a “sit and wait” foraging behavior as well as an active foraging mode depending on the season and hence food availability, and its diet varies from insects to seeds and fruits, and many other species they can capture, even snails and young snakes [35,36,37]. Two morphological subspecies *L. s. stellio* and *L. s. daani* co-exist in the Cyclades: the former inhabiting Delos and the Mykonos archipelago, and the latter being present in Naxos, Paros, and Antiparos. This co-existence in neighboring islands is explained by ancient, human-mediated transport rather than dispersal [38]. The presence of newly-discovered populations in Corfu (Ionian Sea, Greece) [39,40], Karpathos [41] and Crete [42] highlight the role of humans in shaping the present-day distribution of this species. 

Fieldwork was conducted in May, July, and October 2017 in Naxos (Aegean Sea, Greece). In each season, lizards were captured in the stone walls surrounding Demetra’s temple in Sagri (37.029° N, 25.431° E). This area is covered by low vegetation, mainly phrygana and maquis, and extensive stonewalls serve as a refuge for this species, as well as other reptiles [43]. Naxos has a typical Mediterranean climate according to the Koppen/Geiger climate classification, characterized by long dry summers and mild winters, and strong winds blowing during the whole year [44]. Accordingly, precipitation is fairly low (400 mm/yr on average) and mostly occurs between autumn and spring [45].

### 2.2. Operative (Te) and Body (Tb) Temperatures

Operative temperatures (Te) sketch out the thermal environment in which lizards live, as they correspond to the body temperatures that animals would achieve if they didn’t make any effort to actively thermoregulate [46]. To evaluate operative temperatures in autumn, spring, and summer, we used copper models sealed with plasticine and containing 2.5–3 mL of water inside. Previous research suggests that the temperatures monitored by these models as well as their heating/cooling rates showed a strong linear correlation with those exhibited by living individuals, and therefore, match the focal species’ thermal capacity [47,48]. In each season, measurements were recorded every 30 min for three consecutive days, using 20 models that were connected to five data loggers (HOBO U12 4—Channel External Data Logger—U12—008; [49]) (Table 1). To sample all of the microhabitats shaping the thermal niche of the species, models were randomly placed under full sunlight (e.g., lying on a stonewall), in the shade (e.g., inside crevices), and in semi-light exposure (e.g., on the side of a stonewall or a bush) [50].

In each season, body temperatures (Tb) were measured in wild-caught males (with SVL > 85 mm, which is the typical adult size [51]), immediately after capture [52,53] by inserting a type K thermocouple, connected to a mini-logger (EasyLog—USB—1, Lascar Electronics Ltd., Whiteparish, UK), directly into the cloaca [54]. A total of 22 males were caught in spring, 30 in summer and 26 in autumn. Females were excluded from the study to avoid physiologically triggered Tb changes due to gravidity [55]. SVL was measured with a digital caliper (Silverline 380244, accurate to 0.01 mm) and weight with a digital balance (0.0001 g precision) (Table 1). 

### 2.3. Preferred Temperatures (Tpref)

In each season, a subset of lizards captured in the field for Tb measurements were subsequently transferred to the laboratory facilities of the Department of Biology at the National and Kapodistrian University of Athens, where they were housed in individual terraria (60 cm × 30 cm × 40 cm). Each terrarium contained sand as substrate and a tile that served as an artificial shelter as well as a basking spot, allowing lizards to behaviourally thermoregulate under a 60 W incandescent heating lamp (operating 8 h/day). Lizards were fed every other day with mealworms (*Tenebrio molitor*) coated with a multivitamin powder (TerraVit Powder, JBL GmbH and Co. KG, Neuhofen, Germany) and water was provided ad libitum. Sunlight entering through two 2.5 m × 1.5 m windows allowed for a natural photoperiod. A continuously operating air conditioning system kept the room temperature at 25 °C.

The most reliable way to determine an organism’s preferred temperature is by observing its body temperature in controlled thermal environments, which typically feature connected compartments or gradients, and allow the organism to select its desired temperature [5]. Thus, preferred temperatures (Tpref), were estimated for each male (N = 13 in spring, N = 11 in summer, and N = 8 in autumn). Each individual was allowed to thermoregulate within a thermal gradient, ranging from 15 to 60 °C (Table 1). To create this gradient, two heating lamps (100 W 4 and 60 W), and two ice bags were positioned at the opposite sides of a terrarium (100 cm × 25 cm × 25 cm) [56]. Body temperatures were measured again using the same type of K thermocouple previously described, but this time it was taped on the animal’s back (without impeding locomotion) so that it could remain inside the cloaca for the whole duration of the experiment, minimizing the stress on the animal caused by handling that could potentially affect its body temperature [57]. Each lizard was allowed to thermoregulate for an hour prior to the beginning of the experiment [55,58]. Measurements were recorded every five minutes for a period of 5 h (from 10:00–15:00). The interquartile range (middle 50%) of the preferred body temperatures (Tpref) of each individual [4] was used to estimate the set-point temperature range (Tset) in each season, with the average values setting the upper and lower limits of Tset (Table 1).

### 2.4. Effectiveness of Thermoregulation 

The effectiveness of thermoregulation (E) was estimated using the widely used E index [4] which is based on the ability of an animal to achieve body temperatures (Tb) within the range of its thermal preference (Tset), and the degree to which this is enabled or impeded by the thermal habitat (Te). This interrelation is depicted in the Hertz index: E = 1—(db/de), where db is the mean deviation (absolute values) of Tb from Tset, while de is the mean deviation (absolute values) of Te from Tset. Hence, db alone shows the accuracy of thermoregulation while de reveals the thermal quality of a particular habitat (Table 1). Taken together, these deviations point to the active effort made by an animal to thermoregulate effectively. Therefore, E values close to 0 correspond to thermoconformers, animals that select a microhabitat randomly, while values close to 1 describe thermoregulators: animals actively selecting a microhabitat that is appropriate for thermoregulation [4]. 

### 2.5. Statistical Analyses 

All data were log10 transformed to meet the assumptions of parametric analyses after testing for normality and homogeneity of variances. Differences in operative temperatures, de, and db were explored using one-way Analysis of Variance (ANOVA). Important statistical differences were identified using Tukey’s HSD post-hoc test. Seasonal differences regarding field-measured body temperatures and lab-measured preferred temperatures (using individual mean values) were explored using a one-way Analysis of Covariance, with weight and SVL as covariates. The effectiveness of thermoregulation (E) was estimated using a bootstrap resampling method, with the E index and its 95% confidence intervals being calculated by 1000 replicates [4]. Differences among seasons were again identified using Tukey’s HSD post hoc test with *p* ≤ 0.05. All statistical analyses were performed in SPSS Statistics version 27.0.1.0 (IBM 2020, Armonk, NY, USA).

## 3. Results

### 3.1. Thermal Measurements (Te, Tb, Tpref)

Mean values, ranges, and sample sizes for all thermal measurements are presented in Table 1. Mean operational temperatures differed significantly among all seasons, with the highest temperatures being recorded in summer, the lowest in autumn, and intermediate values in spring (ANOVA, F_2, 4514_ = 669.29, *p* < 0.001, Tukey’s HSD test, *p* < 0.05 in all cases). The opposite pattern of significant differences was observed in the deviation of Te from the preferred temperature range, (de), with the highest mean value observed in October, the lowest in July, and an intermediate value in April (ANOVA, F_2, 4514_ = 2208.5, *p* < 0.001, Tukey’s HSD test, *p* < 0.05 in all cases).

Body temperatures measured in the field were similar across seasons (*p* = 0.945). The same is true for both the animal weight (*p* = 0.882) and SVL (*p* = 0.481). The accuracy of thermoregulation was lower in October when db obtained its maximum value. This difference in db between autumn and the other two seasons was found to be statistically significant (ANOVA, F_2, 75_ = 22.027, *p* < 0.001, Tukey’s HSD post-hoc test, *p* < 0.05). This deviation was minimal in both summer and spring when db was close to zero (Table 1). 

Lizards selected significantly lower Tpref in the summer, higher temperatures in the autumn, and intermediate temperatures in the spring (Table 1), (ANCOVA, F_2, 27_ = 15.290, *p* < 0.001). Tukey’s HSD post-hoc tests indicated significant differences in Tpref between summer and all other seasons (*p* < 0.05), but not between spring and autumn (*p* = 0.123). The breadth of the set-point range doubled during summer.

### 3.2. Effectiveness of Thermoregulation

The effectiveness of thermoregulation differed across seasons according to the bootstrap resampling method. Animals were able to thermoregulate more effectively in the summer (E = 0.97), and less in autumn (E = 0.81) (Table 1). In spring, E was also quite high (E = 0.92). All the aforementioned differences were statistically significant (ANOVA, F_2, 2997_ = 7159.8, *p* < 0.001, Tukey’s HSD post-hoc test, *p* < 0.05 in all cases).

## 4. Discussion

Insular lizards are expected to expend less energy to achieve effective thermoregulation when compared to their mainland counterparts as a result of the milder insular climate they experience [23]. Indeed, the challenging climate of the mainland is often reflected in the effectiveness of thermoregulation: lizards from higher altitudes and mountain ranges on the mainland are often moderate thermoregulators, such as *Liolaemus tandilensis*, (E = 0.50–0.69), *Iberolacerta aurelioi* (E = 0.74–0.83), and *Abronia taeniata* (E= 0.60) [59,60,61]. On the other hand, not all islands are characterized by equally favorable climates due to their discrete morphological attributes such as size, wind exposure, and geographic location [62]. Therefore, lizards inhabiting minuscule islets have to demonstrate exceptional thermoregulation efficiency to ensure their survival [15,26,27,28]. According to our results, this is also the case for *L. stellio* inhabiting the largest of the Cyclades, Naxos Island. The effectiveness of thermoregulation was maximum in the summer (E = 0.97), quite high in spring (E = 0.92), and lower in autumn (E = 0.81). These findings refute our initial hypothesis, stating that the benign climate of a larger island would allow lizards to survive with a lower thermoregulation effort across different seasons. In contrast, temperature fluctuations had a significant effect on the thermoregulation efficiency achieved by this species, which responded swiftly to the environmental challenges posed by seasonality, to ensure its survival. 

Operative environmental temperatures followed the predictable fluctuations of the temperate climate. They were higher in summer, intermediate in spring, and lower in autumn (Table 1). From the animal’s perspective, the thermal quality of their habitat changed accordingly, as the environmental temperatures’ deviation from the Tset range (de) was minimum in July and maximum in October. This seasonal variation in temperature, along with other predictable changes in abiotic factors such as wind intensity, and sunlight exposure, can have a profound effect on lizard physiology and behavior, and hence thermoregulation [5,6,13]. Indeed, summer, as largely expected, provided the most advantageous conditions for precise thermoregulation [20].

The seasonal variation in operative temperatures was not followed by body temperatures (Tb), which remained surprisingly constant. As ectotherms, lizards respond to climatic fluctuations in an effort to maintain their body temperatures within a narrow margin of preferred temperatures, so that they are able to exploit resources and optimize fitness and performance [5,63,64,65,66]. Indeed, body temperatures (Tb) of *L. stellio* were remarkably stable across seasons (fixed around 34 °C, Table 1), and did not follow the seasonal weather variation. This is the opposite pattern of what has been observed in many lacertids, in which Tbs were significantly higher in summer and in spring than in winter [16,29,30,67]. Moreover, lizards were able to thermoregulate with greater accuracy in summer and in spring, as expressed by the extremely low db values, which were close to zero. Accuracy of thermoregulation was much lower in autumn when mean db was quite high (db = 2.4), but lizards still managed to maintain high Tbs. This finding is in accordance with previous research on the behavioral thermoregulation patterns of the closely related *Laudakia* species inhabiting Israel and Egypt, which can achieve body temperatures above the environmental ones [68,69].

The ability of lizards to achieve highly constant temperatures across seasons can be attributed to (a) an adjustment in their thermoregulatory behavior, such as changes in their activity period [63,70], their posture while basking, and/or microhabitat selection and use [71,72], (b) the acclimation of their thermal physiology, i.e., a shift in preferred temperatures [5,73], or (c) a combination of the above. Although changes in microhabitat selection and activity period were not evaluated in the present study, they could contribute to the thermoregulation effort exhibited by *L. stellio* across seasons. Previous studies on closely related *Laudakia* species have shown that they occupy a wide thermal niche breadth, and they can achieve elevated body temperatures through conductive basking, and by shuttling between warmer and cooler microhabitats [68,69]. Apart from the aforementioned behavioral tactics that contribute to the effectiveness of thermoregulation, our study revealed a seasonal shift in preferred temperatures.

**Figure 1:**
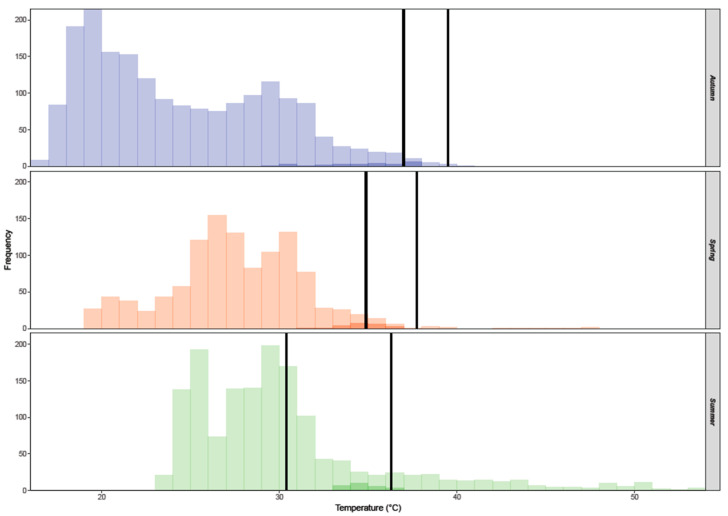
Frequency of field body temperatures (Tb, dark colors) and operative temperatures (Te, light colors) in autumn (blue), spring (orange), and summer (green). Vertical black solid lines indicate the set-point range temperatures (Tset).

The profound effect of seasonality was observed on both mean values, as well as set-point ranges (Tset) of preferred temperatures (Tpref). Lizards selected significantly higher temperatures in October (38.1 °C), lower temperatures in the hot summer month of July (33.2 °C), and intermediate values in May (36.2 °C). The same tendency was observed in Tset (Figure 1), which was shifted from lower temperatures in spring (34.9–37.7 °C) towards higher temperatures in autumn (37.0–39.5 °C). Furthermore, its breadth doubled in summer (30.4–36.3 °C) and so animals could exploit a greater range of available environmental temperatures, as a greater proportion of Te fell within the Tset point range (Figure 1). A shift in preferred temperatures has been reported in many lizard families, such as skinks and agamids [74,75]. In many lacertids, this shift is often a response to seasonality [18,19,26,29,67,76], however, in many of these cases lizards preferred higher temperatures in the summer in comparison to spring. In the case of *L. stellio*, the lower Tpref values observed in summer could reflect the need to avoid overheating and prevent dehydration [30,77] as extremely high temperatures were recorded in July (Figure 1). The Cyclades complex is fairly dry during summer and has one of the highest numbers of consecutive dry days per year in the country [78]. Likewise, a recent study comparing three insular Greek populations of *L. stellio* with *L. cypriaca* from Cyprus showed that the latter opted for much lower temperatures, probably as an adaptation to the extremely hot summers in Cyprus [48].

This shift in thermal preferences could be a response to the seasonality of temperate climate. Shifts in thermal preferences could facilitate thermoregulation effectiveness, by reducing the deviation of Te from Tset. Or they could simply reflect the optimal temperature range for another physiological process, such as sprint speed or digestion [2,19,63]. In general, the extent of behavioral thermoregulation is determined by the trade-off between maximizing physiological performance and individual fitness, and the relevant energy and time constraints, as well as predation risk [10,11]. In our study, we could argue that this shift in Tpref and Tset favored thermoregulation efficiency as well as thermoregulation accuracy, mainly in the summer, by reducing both de and db, and hence the energetic cost of thermoregulation. Given the limitations of this study in terms of sample sizes and the fact that it covers only a single year, the only assumption we could make is that this response is plastic rather than a “fixed” adaptation, as this would require further research spanning multiple seasons and years and including several other insular populations.

Our initial hypothesis suggested that the particularities of insular life such as the low predation pressure [79,80], and the favorable climate [21,22] would lower the costs of thermoregulation. Indeed, the thermoregulation efficiency of many Mediterranean lacertids is higher in the summer than in the spring, mostly driven by the availability of higher environmental temperatures [20]. The favorable climatic conditions, together with the ecological release from predators and competition, allow island species to exploit their thermal habitats more efficiently and thus enjoy greater fitness benefits, which in turn may hinder rather than speed up the evolution of physiology in insular environments [81]. However, the positive effect of the mild island climate appears to be buffered by seasonality, which exerts a stronger effect on thermoregulation in comparison to climate and habitat type [20]. The present study demonstrates this effect, as the most decisive thermal parameter, Tpref, showed substantial seasonal variation and led to the discrepancy in the effectiveness of thermoregulation across seasons. Thus, despite the greater availability of suitable thermal habitats found on larger islands, lizards still face the challenges posed by climatic fluctuations and have to actively thermoregulate to overcome them and ensure their survival. 

Finally, understanding these shifts in thermal physiology will provide valuable insights into the adaptive and evolutionary potential of lizards, which is crucial in view of the ongoing climate change. Lizard species may need to either avoid rising temperatures by moving to more favorable thermal environments, or employ behavioural and physiological plastic mechanisms, or adaptation in order to survive [82,83,84]. While mainland populations can potentially shift their geographic distribution, lizards with limited dispersal abilities, or those occupying a limited space, such as the insular endemics, are often more vulnerable and face a greater risk of extinction [85,86]. This study highlights a shift in the thermal preferences of a Mediterranean lizard during the hot summer period, which could potentially have a buffering effect against the rising temperatures. However, the extent to which this shift can ensure the future survival of the species remains unclear. Thus, effective conservation planning and management should also consider the capacity of species to adapt to these physiological challenges imposed by climate change [87,88].

## 5. Conclusions

This study emphasizes how seasonality impacts the thermal biology of an insular lizard population. During the summer, when the environment provides the best thermal conditions, these lizards can utilize a wider range of temperatures. However, in autumn, the thermal quality of the environment is lowest. Additionally, the efficiency of thermoregulation is influenced by the changing environmental temperatures throughout the seasons, with the highest levels occurring in summer and the lowest in autumn. Furthermore, lizards respond to these seasonal fluctuations by adjusting their thermal preferences, which allows them to maintain consistent body temperatures across seasons. Our findings stress the need for further studies that span across seasons and encompass several insular populations, in order to tease apart the plastic responses and the fixed local adaptations in the thermal biology of these lizards.

## Figures and Tables

**Table 1:** Thermal metrics (Te, de, Tb, db, Tpref, Tset) and thermoregulation effectiveness index (E) across seasons.

Season		Te	de	Tb	db	Tpref	Tset	E
Spring	Mean ± SD	27.7 ± 3.9	7.3 ± 3.6	34.7 ± 1.3	0.6 ± 0.9	36.2 ± 1.2		0.92
	N	1140	1140	22	22	13		
	Range	19.3–47.5	0.0–15.6	31.8–37.0	0.0–3.1	33.5–37.7	34.9–37.7	
	Lower–Upper Q	25.6–30.3	4.6–9.3	34.0–35.8	0–0.9	35.7–36.9		
Summer	Mean ± SD	30.4 ± 5.0	2.8 ± 3	34.3 ± 1.2	0.1 ± 0.3	33.2 ± 2.9		0.97
	N	1490	1490	30	30	11		
	Range	23.8–54.0	0.0–17.7	29.8–36.2	0.0–1.8	27.1–36.7	30.4–36.3	
	Lower–Upper Q	26.0–31.7	0.2–5.0	33.9–35.8	0–0	32.5–35.6		
Autumn	Mean ± SD	24.4 ± 5.2	12.6 ± 5.1	34.8 ± 2.5	2.4 ± 2.4	38.1 ± 0.6		0.81
	N	1881	1881	26	26	8		
	Range	16.8–40.3	0.0–20.2	30.0–38.0	0.0–7.0	36.7–39.0	37.0–39.5	
	Lower–Upper Q	19.9–28.7	8.3–17.1	33.0–37.0	0.0–4.0	37.6–38.8		

## Data Availability

All data in this study are available upon request from the authors and will be made publicly available pending publication of additional manuscripts.
